# Effect of pressure-controlled ventilation-volume guaranteed mode combined with individualized positive end-expiratory pressure on respiratory mechanics, oxygenation and lung injury in patients undergoing laparoscopic surgery in Trendelenburg position

**DOI:** 10.1007/s10877-021-00750-9

**Published:** 2021-08-26

**Authors:** Jianli Li, Saixian Ma, Xiujie Chang, Songxu Ju, Meng Zhang, Dongdong Yu, Junfang Rong

**Affiliations:** Department of Anesthesiology, Hebei General Hospital, Shijiazhuang, 050051 China

**Keywords:** Pressure-controlled ventilation-volume guaranteed mode, Individualized PEEP, Lung protection, Laparoscopic surgery, Trendelenburg position

## Abstract

The study aimed to investigate the efficacy of PCV-VG combined with individual PEEP during laparoscopic surgery in the Trendelenburg position. 120 patients were randomly divided into four groups: VF group (VCV plus 5cmH_2_O PEEP), PF group (PCV-VG plus 5cmH_2_O PEEP), VI group (VCV plus individual PEEP), and PI group (PCV-VG plus individual PEEP). *P*_mean_, *P*_peak_, Cdyn, PaO_2_/FiO_2_, *V*_D_*/V*_T_, A-aDO_2_ and Qs/Qt were recorded at T_1_ (15 min after the induction of anesthesia), T_2_ (60 min after pneumoperitoneum), and T_3_ (5 min at the end of anesthesia). The CC16 and IL-6 were measured at T_1_ and T_3_. Our results showed that the *P*_mean_ was increased in VI and PI group, and the *P*_peak_ was lower in PI group at T_2_. At T_2_ and T_3_, the Cdyn of PI group was higher than that in other groups, and PaO_2_/FiO_2_ was increased in PI group compared with VF and VI group. At T_2_ and T_3_, A-aDO_2_ of PI and PF group was reduced than that in other groups. The Qs/Qt was decreased in PI group compared with VF and VI group at T_2_ and T_3_. At T_2_, *V*_D_*/V*_T_ in PI group was decreased than other groups. At T_3_, the concentration of CC16 in PI group was lower compared with other groups, and IL-6 level of PI group was decreased than that in VF and VI group. In conclusion, the patients who underwent laparoscopic surgery, PCV-VG combined with individual PEEP produced favorable lung mechanics and oxygenation, and thus reducing inflammatory response and lung injury.

**Clinical Trial registry**: chictr.org. identifier: ChiCTR-2100044928

## Introduction

Laparoscopic surgery has been widely adopted in different surgery fields, due to its advantages such as minimal incision, less stress response and fewer blood loss. During laparoscopic surgeries, CO_2_ pneumoperitoneum combined with Trendelenburg position is commonly used to provide adequate exposure of surgical viewing and space. However, these methods have a major impact on the cardiovascular and pulmonary systems such as mean arterial pressure increased, increased *P*_peak_ and decreased pulmonary compliance, and increases the risk of atelectasis or barotrauma because of abdominal content to move toward the head and forcing the diaphragm to elevate [1]. Moreover, increased airway pressure or excessive tidal volume may do harm to alveolar epithelial cells during mechanical ventilation, which leads to the destruction of lung parenchyma [2]. Above effects may produce serious consequences especially in patients with morbid obese or chronic lung disease [3]. Therefore, it is necessary to seek for appropriate lung-protective ventilation strategies to reduce cardiopulmonary complications for patients undergoing laparoscopic surgery in Trendelenburg position.

Although the volume-controlled ventilation (VCV) is commonly used in general anesthesia, it is still reported to cause volutrauma, barotrauma and uneven gas distribution in the lungs due to offering a high airway pressure [4]. Pressure-controlled ventilation (PCV) achieves the desired tidal volume (V_T_) at lower airway pressure delivered by a decelerating flow. However, it leads to unfixed minute ventilation [5] and provokes lung injury due to a tractive force on alveoli [6]. PCV-VG, a relatively innovative ventilation mode, has recently been introduced in the field of anesthesiology. It has the features of VCV and PCV, which delivers a target V_T_ with a decelerating airflow, reduces high airway pressure-induced airway and alveolar damage and ensures effective alveolar ventilation according to the patient’s lung compliance [7, 8]. In recent years, many researches have shown that PCV-VG provides lower airway pressure and better lung mechanics and exerts a huge potential lung-protective effect in various fields [8–10]. Additionally, numerous studies also showed that lung mechanics and gas exchange can be improved by the application of PEEP [10–13]. However, it was unreasonable to apply a fixed PEEP for all patients, and it is critical to determine individualized PEEP by the "titration method" to stabilize the lung function and minimize lung injury, thereby contributing to preferable physiologic and lung-protective effects [10, 14]. The optimal PEEP titration determined by Cdyn is relatively simple and practical, which has been proven to reduce the incidence of postoperative respiratory complications (PPCs) in patients with abdominal surgery [13].

Our previous research had shown that the ventilation strategy of PCV-VG plus individualized PEEP during one-lung ventilation exerted lung-protective effects, as indicated by improved respiratory mechanics, favorable ventilation efficiency and reduced inflammation response [10]. Nevertheless, whether the application of PCV-VG together with individualized PEEP can provide the lung-protective effect in patients undergoing laparoscopic surgery in the Trendelenburg position is unclear. In this randomized study, we investigated the efficacy of PCV-VG together with individualized PEEP on lung mechanics, oxygenation parameters and lung injury in patients underwent laparoscopic surgery in the Trendelenburg position.

## Materials and methods

### Study design

This study was approved by the Ethics Committee for Clinical Trial of Hebei General Hospital, China (ethics approval no.2019–48) and performed at the department of anesthesiology from September 2020 to February 2021. Each patient or family member signed informed consent. The clinical trial registration number was ChiCTR2100044928. The trial enrolled 140 patients with ASA I-III who underwent laparoscopic surgery in Trendelenburg position. Of these, 20 patients were excluded and a total of 120 patients completed the study. Before the operation, patients with morbid obesity (body mass index > 30 kg/m^2^), hypotension (systolic blood pressure < 100 mmHg), bradycardia (heart rate < 60 bpm), cardiologic disease, hypoxia (PaO_2_ < 60 mmHg or SpO_2_ < 90%), chronic pulmonary disease or lung infection were excluded. Patients who were younger than 20 years or older than 70 years were also excluded. Dropout criteria were a conversion in type of surgery procedure to laparotomy, intraoperative blood transfusion, CO_2_ pneumoperitoneum duration < 60 min or > 180 min. Patients were randomly assigned in a 1:1:1:1 ratio to one of four group using a computerized randomization table by an investigator who was blinded to the group assignment.

### Anesthesia and surgery

After entering the operating room, all patients were monitored by electrocardiograpgy (ECG), heart rate (HR), blood pressure, pulse oxygen saturation (SpO_2_) and bispectral index (BIS). All patients underwent radial artery puncture and catheterization were performed to check the blood gases and continuous hemodynamic monitoring. The anesthesia was performed by the same anesthesiologist. Before induction, all patients were preoxygenated with 100% oxygen for at least 3 min. Tracheal intubation was completed after intravenous injection of etomidate 0.3 mg/kg, sufentanil 0.3 μg/kg, cis-atracurium 0.15 mg/kg and midazolam 0.05 mg/kg. Anesthesia was maintained by continuous intravenous remifentanil and propofol infusion, sevoflurane inhalation and intermittent administration of cis-atracurium to maintain the BIS at 40 to 60.

### Ventilation protocol

**Fig. 1:**
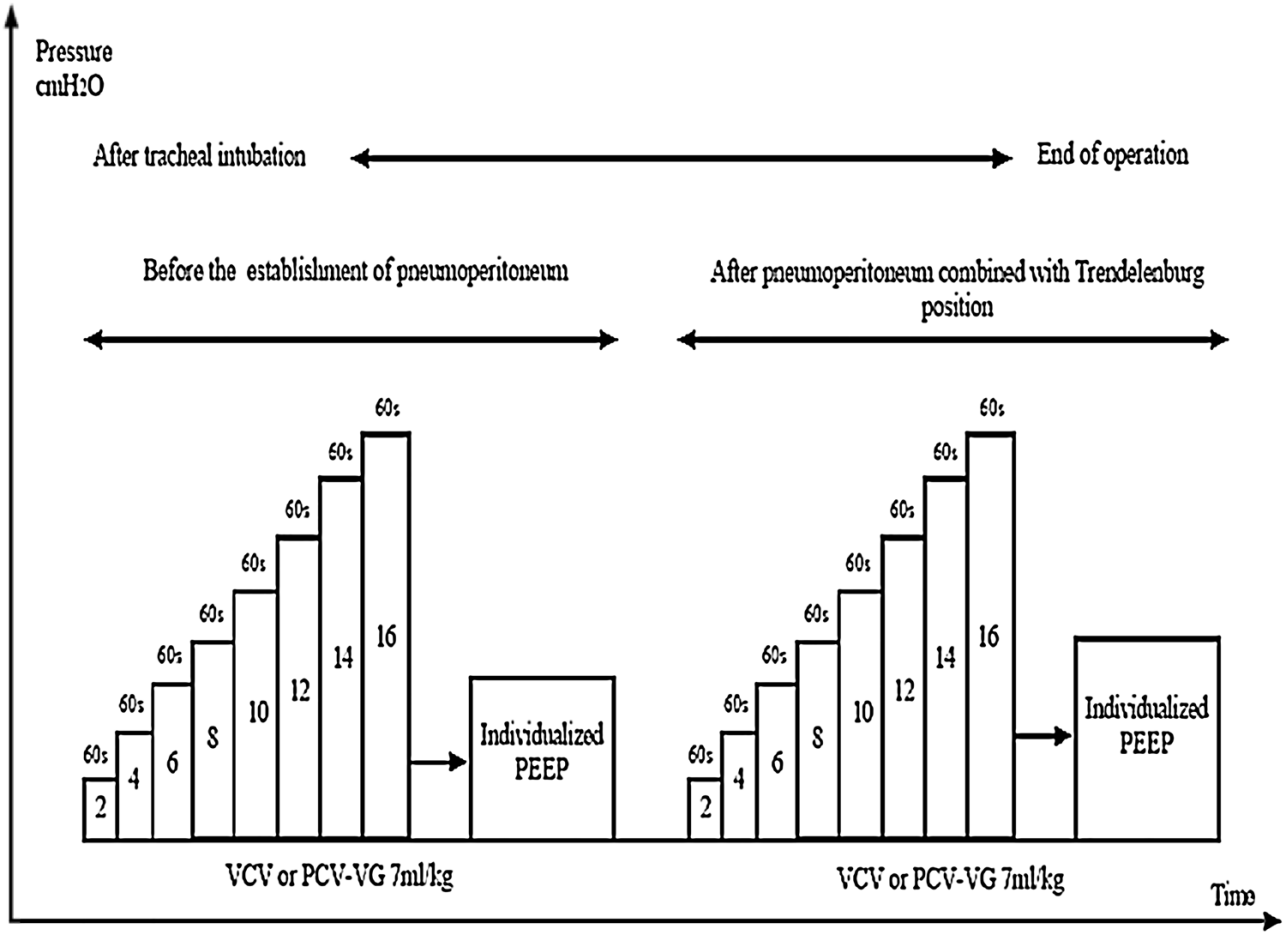
Study protocol of the incremental PEEP titration procedure directed by Cdyn in patients of the VI and PI group

After intubation, all patients in the four groups were ventilated with an anesthesia ventilator (Avance CS2 Pro; GE Healthcare, Piscataway, NJ, USA). The intraperitoneal pressure was adjusted to 12 ± 2 mmHg with CO_2_ insufflation, and then 30°Trendelenburg position was set up. Before surgery, all participants were set the same ventilation parameters, consisting of a fraction of inspired oxygen (FiO_2_) of 0.8, VT of 7 mL/kg PBW, and an initial PEEP of 5 cmH_2_O, which was maintained in the VF and PF group throughout the whole procedure. The I:E ratio was 1:2 and respiratory rate (RR) was adjusted to maintain P_ET_CO_2_ of 35 ± 5 mmHg. The PBW was calculated according to a predefined formula:50 + 0.91 × (centimeters of height-152.4) for men and 45.5 + 0.91 × (centimeters of height-152.4) for women. The incremental PEEP titration [14] was performed two times in VI and PI group. In both groups, the first incremental PEEP titration was performed immediately after intubation, and the individualized PEEP level was set and maintained until the establishment of pneumoperitoneum. The second incremental PEEP titration was performed after the establishment of pneumoperitoneum together with Trendelenburg position, and the individualized PEEP level was set and maintained until the end of pneumoperitoneum together with Trendelenburg position. Also, patients received the individualized PEEP level of the first PEEP titration from the end of pneumoperitoneum together with Trendelenburg position until extubation. The PEEP titration method was as follows (Fig. 1): PEEP was progressively increased by 2 cmH_2_O steps from ZEEP up to 16 cmH_2_O, and each PEEP level was kept for 1 min before measuring Cdyn. The individualized PEEP was considered when the greatest Cdyn was produced.

### Measurements

Researched variables were measured as follows: Blood gas analysis data, P_ET_CO_2_, PEEP, *P*_peak_, *P*_mean_, Cdyn, PaO_2_/FiO_2_, A-aDO_2_, *V*_D_*/V*_T_ and Qs/Qt at T_1_: in the supine position 15 min after the induction of anesthesia, T_2_: 60 min after CO_2_ pneumoperitoneum and Trendelenburg position, and T_3_: 5 min after placement in the supine position at the end of anesthesia.

Parameters were calculated using the following equations:

$$V_{D} /V_{T} = \, \left( {PaCO_{2} - \, P_{ET} CO_{2} } \right)/PaCO_{2}$$

$$Qs/Qt = \left( {PA - aDO_{2} \times 0.0031} \right) \div \left( {PA - DO_{2} \times 0.0031 + 5} \right)$$

$$PA - aDO_{2} = [FiO_{2} \times \left( {P_{B} - P_{H2O} } \right) - PaCO_{2} /RQ - PaO_{2}$$

$$\left( {P_{B} = 760 \, mmHg, \, P_{H2O} = 47 \, mmHg, \, RQ = 0.8} \right)$$

The serum concentration of CC-16 and IL-6 in T_1_ and T_3_ was detected by enzyme-linked immunosorbent assays. Postoperative complications (WBC count, cough, expectoration and fever) in the four groups were recorded during the first 3 days after operation.

### Statistical analyses

Statistical analyses were performed using SPSS version 24.0 (*SPSS Inc.*). Continuous variables are expressed as the mean ± standard deviation (SD) or median (interquartile range, IQR). Normal distribution data were analyzed using the Shapiro–Wilk test. Data with a normal distribution were compared among the four groups using one-way *ANOVA* with *LSD-t* as the post hoc test. Continuous variables with a nonnormal distribution in multiple groups were analyzed by the Kruskal–Wallis test. Categorical variables are described as numbers and were analyzed using the chi-squared test. *p*-values were two-sided and *p* < 0.05 was considered statistically significant difference.

## Results

### Patients enrollment and intraoperative characteristics

**Fig. 2:**
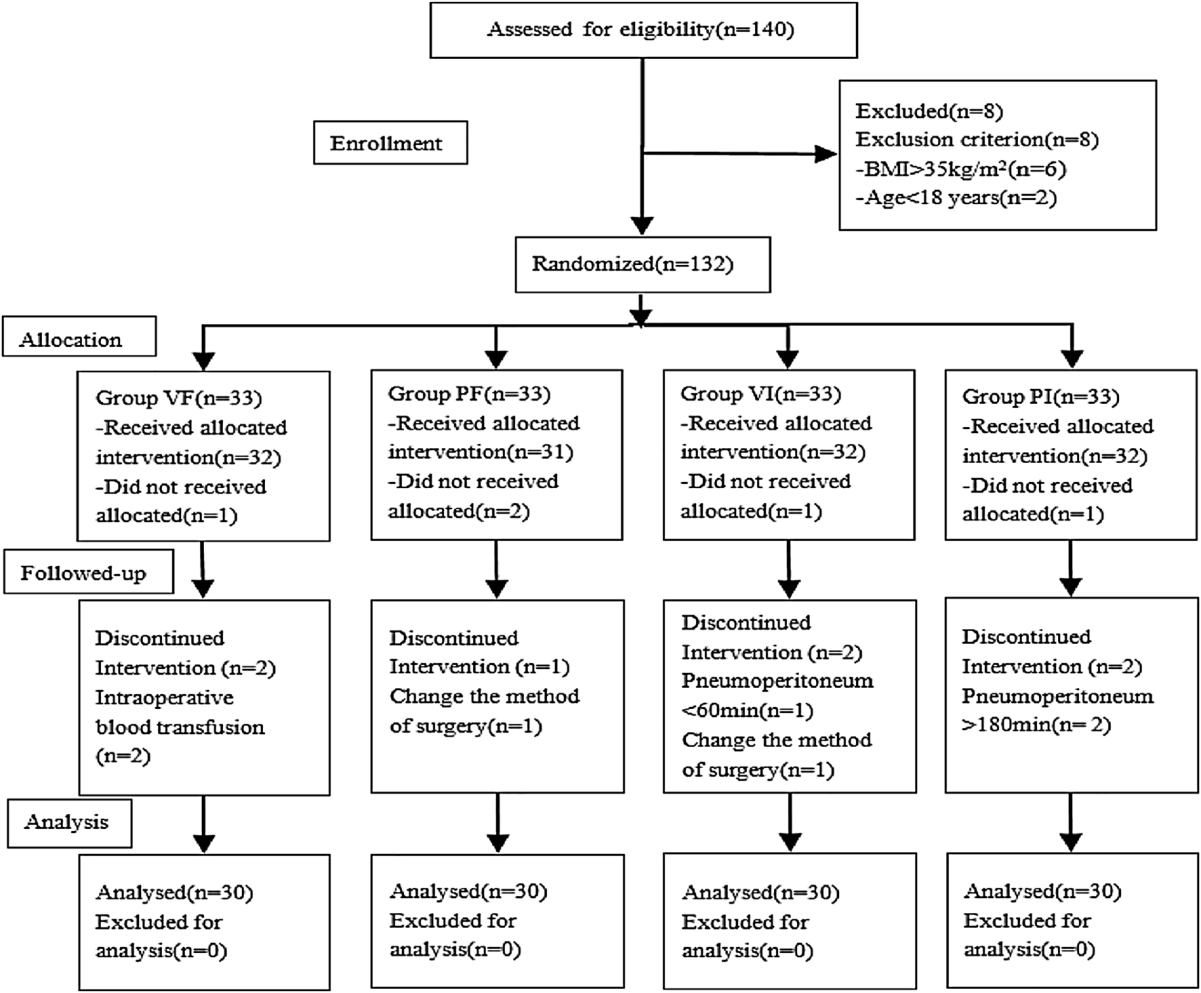
The study protocol

140 patients, who were scheduled to receive laparoscopic surgery in Trendelenburg position, were initially enrolled and follow-up of patients was provided in Fig. 2. Of these, 20 patients were excluded and a total of 120 patients completed the study. There was no significant difference among the groups in terms of characteristics and intraoperative data. (*p* > 0.05) (Tables 1 and 2).

**Table 1:** Demographic Characteristics

Index	VF (n=30)	PF (n=30)	VI (n=30)	PI (n=30)
Age(years)	45.0 ± 14.3	43.9 ± 13.8	48.8 ± 15.6	48.9 ± 13.5
Sex(F/M)	25/5	26/4	25/5	25/5
Height(cm)	162.9 ± 6.8	163.3 ± 7.4	162.2 ± 6.9	162.0 ± 6.8
BMI (kg/m^2^)	24.8 ± 3.6	24.6 ± 3.1	24.7 ± 3.9	25.0 ± 3.0
PBW (kg)	55.1 ± 6.3	55.1 ± 6.0	54.5 ± 6.4	54.3 ± 6.3
ASA(I/II/III)	0/29/1	0/28/2	0/27/3	0/28/2

Data are presented as mean ± standard deviation or number of patients; ASA: American Society of Anesthesiologists, BMI: body mass index, F:female, M: male, PBW: estimated weight; VF: VCV plus fixed PEEP of 5cmH_2_O, PF: PCV-VG plus fixed PEEP of 5cmH_2_O, VI: VCV plus individual PEEP, PI: PCV-VG plus individual PEEP

**Table 2:** Intraoperative Data

Index	VF (n = 30)	PF (n = 30)	VI (n = 30)	PI(n = 30)
Type of surgery				
Gastrointestinal Surgery	8(26.7%)	9(30.0%)	7(23.3%)	8(26.7%)
Gynecology	22(73.3%)	21(70.0%)	23(76.7%)	22(73.3%)
Vasoactive drugs	9(30.0%)	10(33.3%)	8(26.7%)	11(36.7%)
Volume of fluid (mL)	1000(1000,1100)	1000(1000,1500)	1000(1000,1500)	1000(1000,1500)
Urine output (mL)	100(50,200)	100(50,200)	100(50,150)	100(50,100)
Duration of operation (min)	82(70,103)	81(75,107)	95(76,106)	95(90,105)
Duration of anesthesia (min)	112(87,135)	114(100,141)	130(105,155)	130(110,140)
Duration of pneumoperitoneum (min)	68(60,90)	70(65,93)	85(65,100)	85(79,100)
Blood loss (mL)	15(5,23)	20(10,50)	20(10,80)	30(9,50)
HR (bpm)				
T_1_	66.0 ± 9.3	66.3 ± 11.2	67.1 ± 7.7	65.8 ± 6.2
T_2_	75.6 ± 10.9	73.6 ± 7.2	74.6 ± 4.6	75.0 ± 7.0
T_3_	69.2 ± 9.6	67.7 ± 9.0	67.5 ± 5.1	67.6 ± 6.3
MAP (mmHg)				
T_1_	88.8 ± 12.4	82.9 ± 18.1	82.1 ± 14.5	85.3 ± 11.7
T_2_	96.6 ± 17.7	94.9 ± 13.4	95.5 ± 13.5	94.6 ± 15.1
T_3_	87.8 ± 16.6	89.5 ± 14.1	87.0 ± 11.5	87.4 ± 10.6

Data are expressed as mean ± standard deviation (SD) or median (interquartile range).VF: VCV plus fixed PEEP of 5cmH_2_O, PF:PCV-VG plus fixed PEEP of 5cmH_2_O, VI:VCV plus individual PEEP, PI:PCV-VG plus individual PEEP.T_1_:in the supine position 15 min after the induction of anesthesia, T_2_:60 min after CO_2 _pneumoperitoneum and Trendelenburg position, T_3_: 5 min after placement in the supine position at the end of anesthesia

### Respiratory mechanics

There was no statistically significant difference among the groups in the values of tidal volume (*p* > 0.05). Compared with the group VF and PF, the level of PEEP was higher in the group VI and PI at T_1_, T_2_ and T_3_ (*p* < 0.05). At T_2_, the *P*_mean_ was increased in the group VI and PI than that in the group VF and PF (*p* < 0.05). Compared with the group VF, PF and VI, the *P*_peak_ was decreased in the PI group at T_2_ (*p* < 0.05). The Cdyn was higher in the PI group than that in the group VF and PF throughout the study period (*p* < 0.05), and it was increased in the PI group compared with VI group at T_2_ and T_3_ (*p* < 0.05). Also, the Cdyn of the group VI and PF was better than that in the VF group at T_3_ (*p* < 0.05) (Table 3).

**Table 3:** Respiratory mechanics, Ventilatory and Oxygenation parameters

Index	Time Point	VF (n = 30)	PF (n = 30)	VI (n = 30)	PI (n = 30)
V_T_ (mL)	T_1_	386 ± 44	386 ± 42	382 ± 45	383 ± 43
	T_2_	384 ± 44	384 ± 42	381 ± 45	381 ± 44
	T_3_	389 ± 43	384 ± 42	382 ± 43	378 ± 44
PEEP (cmH_2_O)	T_1_	5	5	6(4,8) ^▲△^	6(4,8) ^▲△^
	T_2_	5	5	8(6,10) ^▲△^	8(6,10) ^▲△^
	T_3_	5	5	6(4,8)^▲△^	6(4,8) ^▲△^
*P*_peak_ (cmH_2_O)	T_1_	15.4 ± 1.9	15.5 ± 2.3	15.9 ± 1.9	15.2 ± 3.2
	T_2_	24.1 ± 2.4	23.4 ± 2.3	24.9 ± 2.5^△^	21.9 ± 2.5^▲△*^
	T_3_	15.6 ± 2.4	15.2 ± 2.7	17.8 ± 2.4^▲△^	15.1 ± 2.9^*^
*P*_mean_ (cmH_2_O)	T_1_	11.0 ± 3.2	10.0 ± 2.2	10.1 ± 3.4	9.8 ± 2.4
	T_2_	13.1 ± 2.2	13.9 ± 2.9	16.5 ± 2.9^▲△^	16.8 ± 2.2^▲△^ 20^▲△^
	T_3_	10.8 ± 1.6	11.6 ± 2.3	10.0 ± 2.0^△^	9.4 ± 2.4^▲△^
Cdyn (mL/cmH_2_O)	T_1_	49.8 ± 8.7	51.2 ± 8.8	54.5 ± 9.9	59.7 ± 16.3^▲△^
	T_2_	25.5 ± 3.7	27.4 ± 4.6	29.0 ± 5.0	33.7 ± 7.1^▲△*^
	T_3_	34.3 ± 3.8	42.1 ± 7.0^▲^	43.6 ± 8.0^▲^	53.2 ± 13.1^▲△*^
PaO_2_/FiO_2_ (mmHg)	T_1_	374 ± 16	378 ± 31	378 ± 18	379 ± 23
	T_2_	278 ± 28	332 ± 39^▲^	298 ± 35^▲△^	338 ± 25^▲*^
	T_3_	286 ± 31	326 ± 14^▲^	289 ± 30^△^	341 ± 16^▲△*^
*V*_D_*/V*_T_	T_1_	0.36 ± 0.03	0.36 ± 0.03	0.37 ± 0.02	0.37 ± 0.02
	T_2_	0.46 ± 0.03	0.43 ± 0.02^▲^	0.44 ± 0.02^▲^	0.34 ± 0.05^▲△*^
	T_3_	0.35 ± 0.03	0.35 ± 0.03	0.36 ± 0.02	0.36 ± 0.07
Qs/Qt	T_1_	0.12(0.12,0.13)	0.12(0.11,0.13)	0.12(0.12,0.13)	0.12(0.11,0.13)
	T_2_	0.16(0.15,0.16)	0.14(0.13,0.16)^▲^	0.15(0.14,0.16)^▲△^	0.13(0.13,0.14)^▲*^
	T_3_	0.15(0.15,0.16)	0.14(0.13,0.14)^▲^	0.15(0.15,0.16)^△^	0.13(0.13,0.14)^▲△*^
A-aDO_2_ (mmHg)	T_1_	226.8 ± 14.3	222.4 ± 26.7	224.0 ± 14.8	223.5 ± 18.6
	T_2_	298.9 ± 21.6	255.6 ± 32.3^▲^	282.8 ± 28.1^▲△^	250.9 ± 20.0^▲*^
	T_3_	291.5 ± 24.5	260.0 ± 13.3^▲^	289.1 ± 22.9^△^	248.1 ± 13.9^▲△*^

Data are expressed as mean ± standard deviation (SD) or median (interquartile range). VF: VCV plus fixed PEEP of 5cmH_2_O, PF: PCV-VG plus fixed PEEP of 5cmH_2_O, VI: VCV plus individual PEEP, PI: PCV-VG plus individual PEEP.T_1_: in the supine position 15 min after the induction of anesthesia, T_2_: 60 min after CO_2_ pneumoperitoneum and Trendelenburg position, T_3_: 5 min after placement in the supine position at the end of anesthesia

Compared with group VF, ^▲^*p* < 0.05

Compared with group PF,^△^*p* < 0.05

Compared with group VI, *p < 0.05

### Ventilation efficiency variables

PaO_2_/FiO_2_ was increased in group PF and PI than that in the VF group at T_2_ and T_3_ (*p* < 0.05), and it was better in the PI group than in the VI group at T_2_ and T_3_ (*p* < 0.05), Also, compared with the VF group, PaO_2_/FiO_2_ was increased in the VI group at T_2_ (*p* < 0.05). At T_2_ and T_3_, A-aDO_2_ of the group PI and PF was reduced compared with the group VF and VI (*p* < 0.05), and it was lower in the VI group than that in the VF group at T_2_ (*p* < 0.05). The Qs/Qt was decreased in the PI group, compared with the group VF and VI at T_2_ and T_3_ (*p* < 0.05), and it was lower in the PF group than that in the VF group (*p* < 0.05). Meanwhile, *V*_D_*/V*_T_ in the PI group was decreased compared with other three groups at T_2_ (*p* < 0.05), and it was increased in the VF group than that in the group PF and VI (*p* < 0.05) (Table 3).

### Blood gas analysis

PaO_2_ was increased in the PI group compared with the group VI and VF at T_2_ and T_3_ (*p* < 0.05). Also, it was increased in the PF group compared with the VF group at T_2_ and T_3_ (*p* < 0.05). At T_2_, PaO_2_ in the VI group was increased than in the VF group (*p* < 0.05) (Table 4).

**Table 4:** Blood gas analysis

Index	Time point	VF (n = 30)	PF (n = 30)	VI (n = 30)	PI (n = 30)
	T_1_	7.39 ± 0.04	7.39 ± 0.03	7.39 ± 0.02	7.39 ± 0.02
pH	T_2_	7.38 ± 0.04	7.38 ± 0.04	7.39 ± 0.03	7.39 ± 0.03
	T_3_	7.38 ± 0.04	7.38 ± 0.04	7.39 ± 0.02	7.39 ± 0.04
PaCO_2_ (mmHg)	T_1_	35.4 ± 3.0	36.5 ± 4.1	35.4 ± 4.0	35.1 ± 1.9
	T_2_	39.5 ± 4.1	39.4 ± 4.0	39.2 ± 1.9	39.1 ± 2.3
	T_3_	40.3 ± 2.9	40.0 ± 2.6	39.9 ± 2.9	39.4 ± 3.3
PaO_2_ (mmHg)	T_1_	299 ± 13	302 ± 24	302 ± 14	303 ± 18
	T_2_	222 ± 22	266 ± 31^▲^	239 ± 28^▲△^	271 ± 20^▲*^
	T_3_	229 ± 25	260 ± 12^▲^	232 ± 24^△^	273 ± 13^▲△*^

Data are expressed as mean ± standard deviation (SD) or median (interquartile range). VF: VCV plus fixed PEEP of 5cmH_2_O, PF: PCV-VG plus fixed PEEP of 5cmH_2_O, VI: VCV plus individual PEEP, PI: PCV-VG plus individual PEEP.T_1_: in the supine position 15 min after the induction of anesthesia, T_2_: 60 min after CO_2_ pneumoperitoneum and Trendelenburg position, T_3_: 5 min after placement in the supine position at the end of anesthesia

Compared with group VF, ^▲^*p* < 0.05

Compared with group PF,^△^*p* < 0.05

Compared with group VI, *p < 0.05

### Serum concentration of CC16 and IL-6

At T_3_, the concentration of serum CC16 in the PI group was lower than that in other three groups (*p* < 0.05), and compared with the group VF and VI, the concentration of serum IL-6 was decreased in the PI group (*p* < 0.05) (Table 5).

**Table 5:** Serum CC16 and IL-6 concentrations (ng/ml)

Index	Time point	VF (n = 30)	PF (n = 30)	VI (n = 30)	PI (n = 30)
CC16	T_1_	5.0 ± 1.2	4.9 ± 1.2	5.0 ± 1.3	5.0 ± 1.4
	T_3_	60.6 ± 11.5	53.4 ± 11.1^*▲*^	53.0 ± 11.0^*▲*^	47.1 ± 11.9^▲△^*
IL-6	T_1_	13.5(5.3,58.6)	10.6(4.6,57.5)	10.9(5.3,58.0)	10.0(4.5,57.4)
	T_3_	66.0 ± 17.4	52.2 ± 18.3^*▲*^	62.2 ± 17.1^*△*^	46.0 ± 23.8^▲^*

Data are expressed as mean ± standard deviation (SD) or median (interquartile range). VF: VCV plus fixed PEEP of 5cmH_2_O, PF: PCV-VG plus fixed PEEP of 5cmH_2_O, VI: VCV plus individual PEEP, PI: PCV-VG plus individual PEEP.T_1_: in the supine position 15 min after the induction of anesthesia, T_3_: 5 min after placement in the supine position at the end of anesthesia

Compared with group VF, ^▲^*p* < 0.05

Compared with group PF, ^△^*p* < 0.05

Compared with group VI, *p < 0.05

### Other clinical endpoints

There was no significant difference in WBC count, the incidence of cough, expectoration and fever within 3 days after operation among the four groups (*p* > 0.05) (Table 6).

**Table 6:** Postoperative outcomes

Index	VF (n = 30)	PF (n = 30)	VI (n = 30)	PI (n = 30)
WBC (× 10^9^)	6.2 ± 1.5	5.8 ± 1.1	5.7 ± 1.4	6.0 ± 1.2
neutrophile (× 10^9^)	5.1 ± 1.5	4.8 ± 0.9	4.7 ± 1.2	4.7 ± 1.6
cough	0(0%)	0(0%)	2(7%)	0(0%)
expectoration	1(3%)	1(3%)	0(0%)	0(0%)
fever	1(3%)	1(3%)	0(0%)	0(0%)

Data are expressed as mean ± standard deviation (SD) or median (interquartile range). VF: VCV plus fixed PEEP of 5cmH_2_O, PF: PCV-VG plus fixed PEEP of 5cmH_2_O, VI: VCV plus individual PEEP, PI: PCV-VG plus individual PEEP

## Discussion

The randomized controlled trial observed that PCV-VG ventilation mode combined with individualized PEEP can improve respiratory mechanics and oxygenation, while decreasing lung injury. These results revealed that the PCV-VG ventilation mode combined with individualized PEEP may be beneficial for patients undergoing laparoscopic surgery in Trendelenburg position.

Laparoscopic surgery is performed using mechanical ventilation and CO_2_ pneumoperitoneum on patients in the Trendelenburg position under general anesthesia. However, CO_2_ pneumoperitoneum and the Trendelenburg position have been reported to increase incidence of PPCs [1]. In addition, inappropriate mechanical ventilation settings may theoretically induce VILI even in patients with normal lungs during general anesthesia [15]. Therefore, it is very important for anesthesiologists to implement ideal lung-protective ventilation strategies to reduce lung injury in patients undergoing laparoscopic surgery in Trendelenburg position.

Usually, lung-protective ventilations, which consist of a lower tidal volume (V_T_), higher PEEP and regular alveolar recruitment maneuvers (ARM), are accepted by anesthesiologists as effective ways to improve oxygenation and reduce VILI during surgeries [16]. However, relevant studies have concluded that the optimal PEEP and actual effects of PEEP remains controversial [17, 18]. Fixed PEEP may not fit each patient and proper PEEP regulation may produce significant lung-protective effect, whereas improper PEEP levels may lead to lung tissue hyperinflation or pulmonary atelectasis [19, 20]. Therefore, it is important to determine individualized PEEP to suit the individual lung physiology of different patient. A recent study showed that an optimal individualized PEEP level determined by a static pulmonary compliance-directed PEEP titration was superior to conventional ventilation mode and exerted a favourable lung-protective effect during general anesthesia in laparoscopic total hysterectomy [12].

At present, the PEEP titration based on lung compliance has two methods: incremental titration and decremental titration. Previous study shown that both the incremental titration and decremental titration were able to decrease intraoperative shunt, but only the decremental titration improved oxygenation and lowered driving pressure [14]. Our previous study suggested that the decremental PEEP titration can improve respiratory mechanics, oxygenation parameters, and the inflammatory reaction during one-lung ventilation [10]. However, another resaerch suggested that the incremental titration can also improve oxygenation [21], which was similar to our present study.

VCV and PCV, commonly used by clinical practice, have some disadvantages in different patterns. PCV-VG is a relatively new ventilation mode, which initially transmits a preset V_T_ by a decelerating flow at a lower airway pressure and automatically adjusts the airway pressure of the next breath by measuring a patient’s inspiratory pressure and pulmonary compliance [22]. Theoretically, PCV-VG is suitable for maintaining an appropriate V_T_ during laparoscopic surgery with Trendelenburg position, where CO_2_ pneumoperitoneum and position adjustments may cause sudden changes in intra-abdominal pressure. Recently, some studies about the lung-protective effect of PCV-VG superior to other ventilation modes were established during laparoscopic surgery [22, 23]. Nevertheless, lung-protective effects of PCV-VG has not been deeply studied, and the efficacy of PCV-VG combined with individualized PEEP on patients undergoing laparoscopic surgery in Trendelenburg position is not known.

CO_2_ pneumoperitoneum and Trendelenburg position leaded to increased intrathoracic pressure, increased *P*_peak_ and decreased lung compliance [8]. The results of the present study are consistent with our expectation that using an individualized PEEP under PCV-VG mode can improve lung mechanics, pulmonary gas exchange, and arterial oxygenation as well as hemodynamic stability. Firstly, our data showed that PCV-VG combined with individualized PEEP produced a lower *P*_peak_, which was in line with our recent data during one-lung ventilation [10]. *P*_peak_ level was probably not accurately reflect alveolar pressure [24], and it might be worthless due to the resistance of the tracheal tube and the ventilation mode-related difference in endinspiratory flow [25]. Nevertheless, *P*_mean_ closely reflects mean alveolar pressure and correlates with alveolar ventilation and gas oxygenation [26]. In the present study, our data indicated that the *P*_mean_ in the PI and VI group was higher than that in the group PF and VF, and the Cdyn in the PI group was increased compared with other three groups, which were consisted with the results of our recent study in one-lung ventilation [10], implying the actions of reasonable individualized PEEP is superior to fixed 5 cmH_2_O of PEEP. Furthermore, it usually requires increased *P*_mean_ by the application of extrinsic PEEP to prevent low V_T_-induced atelectasis and hypoventilation [27]. However, it needs to be noted that an abnormally higher *P*_mean_ may lead to hemodynamic instability. In this study, there was no difference in hemodynamics among the four groups, probably due to the higher *P*_mean_ did not substantially affect hemodynamic stability.

PCV-VG can improve oxygenation and reduce the pulmonary shunt due to decelerating flow and higher *P*_mean_. However, the data from a recent study indicated PCV-VG did not produce better oxygenation than VCV, despite the increased *P*_mean_ and higher Cdyn observed during robot-assisted laparoscopic gynecologic surgery in the Trendelenburg position [8]. Moreover, it requires intraoperative use of PEEP to prevent unwanted pulmonary pathophysiological effects induced by CO_2_ pneumoperitoneum and Trendelenburg position [28]. Hence, we designed this trial to explore the effect of PCV-VG plus individualized PEEP on alveolar ventilation and oxygenation. Our results suggested that PaO_2_/FiO_2_ was increased in the PI group compared with other groups, and the lower A-aDO_2_, Qs/Qt and *V*_D_*/V*_T_ in the PI group were detected in this study, which suggested that PCV-VG combined with individualized PEEP mode resulted in superiority for improving ventilation and oxygenation. It might be associated with the impact of higher *P*_mean_ on oxygenation and the prevention of atelectasis under the automatic adjustment of PCV-VG mode. In fact, decelerating flow of PCV-VG mode with high initial flow velocity is different with the constant flow pattern observed in VCV mode, which contributes to ventilation-perfusion matching and reduces pulmonary shunt [29]. Moreover, optimal PEEP results in minimal dead-space and maximal arterial oxygen tension and compliance. According to our study, 8cmH_2_O might be the optimal PEEP level for patients undergoing laparoscopic surgery in Trendelenburg position, which is consist with a previous study [28]. As a matter of fact, excessively higher *P*_mean_ could result in Qs/Qt disturbance. Nevertheless, the Qs/Qt was lower in the PI group in our study, indicating that acceptable *P*_mean_ made alveoli properly open.

Laparoscopic surgery itself induces increased inflammatory biomarkers after operation despite minimally invasive surgical procedure [12]. Recently, the data of a study from our team showed that the inflammatory mediator neutrophil elastase participated in acute lung injury during one-lung ventilation and the PCV-VG combined with individualized PEEP ventilation strategy exerted a protective effect against lung injury by decreasing neutrophil elastase concentration [10]. CC-16, as an inflammatory mediator, secretes mainly from Clara cells in the airway epithelium of the distal lung. Research has shown that CC-16 may indicate the occurrence of atelectasis and lung hyperinflation in general anesthesia patients undergoing mechanical ventilation [30]. IL-6 is also an early predictor of VILI, reflecting the degree of lung damage and inflammation [31]. In current study, significant differences were obtained in the serum levels of CC16 and IL-6 in the PI group compared with other groups, indicating the ventilation strategy of PCV-VG combined with individualized PEEP could alleviate ventilator-induced inflammatory response and lung injury. However, a recent study showed that the individualized PEEP alone did not make a difference to the inflammatory process compared with conventional ventilation mode in patients undergoing laparoscopic total hysterectomy surgery [12]. It was possible that PCV-VG aside from individualized PEEP was adopted in our study. Unfortunately, we did not found significant differences about the postoperative complications among the groups such as fever, infection, cough and expectoration. Whether the strategy of PCV-VG combined with individualized PEEP could improve postoperative outcomes requires further study using more accurate parameters.

There were some potential limitations in our present study. Firstly, the study population in this research was relatively small and further studies should be conducted to confirm these results at multiple centers. Secondly, our study did not include patients with obesity or respiratory disease, which were important factors for compromising oxygenation and respiratory mechanics.

In conclusion, the ventilation strategy of PCV-VG combined with an individualized PEEP was beneficial to intraoperative respiratory mechanics, oxygenation parameters, and the inflammatory reaction. This ventilation strategy may be a feasible alternative ventilation mode in patients undergoing laparoscopic surgery in Trendelenburg position.

## Data Availability

The clinical data used to support the findings of this study are available from the corresponding author upon reasonable request.
